# Prognostic Value of Tumor Size in Patients with Upper Tract Urothelial Carcinoma: A Systematic Review and Meta-analysis

**DOI:** 10.1016/j.euros.2022.06.001

**Published:** 2022-06-28

**Authors:** Runzhuo Ma, Zenan Liu, Yinchu Cheng, Pengxiang Zhou, Yuting Pan, Hai Bi, Liyuan Tao, Bin Yang, Haizhui Xia, Xuehua Zhu, Jide He, Wei He, Guoliang Wang, Yi Huang, Lulin Ma, Jian Lu

**Affiliations:** a Department of Urology, Peking University Third Hospital, Beijing, China; b Center for Robotic Simulation & Education, Catherine & Joseph Aresty Department of Urology, USC Institute of Urology, University of Southern California, Los Angeles, CA, USA; c Department of Pharmacy, Peking University Third Hospital, Beijing, China; d Department of Epidemiology and Biostatistics, School of Public Health, Peking University, Beijing, China; e Department of Biostatistics, Peking University Third Hospital, Beijing, China

**Keywords:** Meta-analysis, Prognosis, Tumor size, Upper tract urothelial carcinoma

## Abstract

**Context:**

The role of tumor size in predicting prognosis in upper tract urothelial carcinoma (UTUC) patients remains poorly defined.

**Objective:**

To assess the prognostic value of tumor size in patients with UTUC through a systematic review and meta-analysis.

**Evidence acquisition:**

A comprehensive literature search of the PubMed and Embase databases were performed to identify all relevant articles published up to December 2021 according to the Preferred Reporting Items for Systematic Reviews and Meta-analyses (PRISMA) statement. Available hazard ratios (HRs) and corresponding 95% confidence intervals (95% CIs) were analyzed to evaluate the association between tumor size and survival outcomes.

**Evidence synthesis:**

A total of 35 articles representing 32 292 patients met the eligibility criteria and were finally included for the meta-analysis. Tumor size was significantly associated with poor outcomes in terms of overall survival (HR = 1.42, 95% CI = 1.28–1.58), cancer-specific survival (HR = 1.66, 95% CI = 1.47–1.88), recurrence-free survival (HR = 1.25, 95% CI = 1.13–1.38), and intravesical recurrence (HR = 1.12, 95% CI = 1.04–1.20). There was between-study heterogeneity in the effect of tumor size on all these meta-analyses, with *p* < 0.10 and I^2^ generally >50%. Subgroup analyses illustrated that the association of tumor size with adverse prognosis in UTUC patients is not affected by treatment modalities. Segmental resection of ureter, whether receiving lymph node dissection, cutoff of tumor size, and region of population were potential sources of heterogeneity. The funnel plot test indicated no significant publication bias in the meta-analysis of survival outcomes.

**Conclusions:**

This study shows that larger tumor size is associated with an increased risk of overall and cancer-specific mortality, and disease recurrence in UTUC. Integration of tumor size with other prognostic indicators may help in risk stratification and individualized treatment of UTUC.

**Patient summary:**

Through a systematic review and meta-analysis, this study found that larger tumor size is associated with an increased risk of overall and cancer-specific mortality, and disease recurrence in patients with upper tract urothelial carcinoma.

## Take Home Message

Our study summarized the existing evidence through a systematic review and meta-analysis, and found that larger tumor size is associated with an increased risk of overall and cancer-specific mortality, and disease recurrence in upper tract urothelial carcinoma patients.

## 1 Introduction

Upper tract urothelial carcinoma (UTUC) is a relatively uncommon malignancy derived from the urothelium along the pyelocaliceal cavities and ureter, accounting for approximately 5–10% of all urothelial carcinomas [1]. Although a conservative approach to treatment has achieved encouraging results in selected patients, radical nephroureterectomy (RNU) with ipsilateral bladder cuff excision remains the standard treatment for high-risk patients with nonmetastatic disease [2], [3], which has provided durable local tumor control and better long-term survival.

Despite the advancement of surgical techniques and the increasing application of perioperative systemic chemotherapy, survival outcomes of patients with UTUC have not improved significantly over time, with up to 30% of patients, particularly those with advanced disease, experiencing disease recurrence and cancer-specific death [4]. To improve the oncological outcomes of UTUC, prognostic factors have been identified to guide clinical decision-making and risk stratification. However, these factors are mainly pathological features, such as tumor stage and grade, tumor location and architecture, concomitant carcinoma in situ, and lymphovascular invasion (LVI) status [5], [6], which can only be acquired postoperatively.

Tumor size is an essential variable when assessing the characteristics of urothelial carcinoma and can be acquired conveniently in preoperative imaging [7]. Tumor size has been identified as a risk factor for poor oncological outcomes in bladder cancer, while its prognostic impact in UTUC has not been addressed fully. Some studies reported that tumor size larger than 3 or 4 cm was associated with poor overall survival (OS), cancer-specific survival (CSS), recurrence-free survival (RFS), and higher risk of intravesical recurrences (IVRs) after RNU [8], [9]. However, other studies reached diverse conclusions [10], [11]. This is likely due to the limitations of small sample size and the heterogeneity of treatment modalities in these studies.

Therefore, the aim of this study is to conduct a systematic review and meta-analysis to summarize the existing evidence to determine the prognostic value of tumor size in patients with UTUC, and perform subgroup analyses to address the heterogeneity of included studies.

## 2 Evidence acquisition

### 2.1 Search strategy

The present systematic review and meta-analysis was performed according to the Preferred Reporting Items for Systematic Reviews and Meta-analyses (PRISMA) statement [12] and has been registered on PROSPERO (www.crd.york.ac.uk/PROSPERO; CRD42019133468). The PubMed and Embase databases were searched to identify reports published until December 2021 regarding the prognostic value of tumor size in UTUC. The following search terms were used separately or in combinations: (“upper urinary tract” OR “urinary tract” OR “urothelial”) AND (“carcinoma” OR “neoplasms” OR “tumor” OR “cancer”) AND (“tumor size” OR “tumor diameter” OR “tumor volume”) AND (“prognosis” OR “outcomes” OR “survival” OR “prognostic”). Reference lists in the relevant publications were checked for any other potential studies. Initial screening was performed independently by two investigators based on the titles and abstracts to identify ineligible reports, and reasons for exclusions were noted. Potentially relevant reports were subjected to a full-text review, the relevance of the reports was confirmed, and the data were extracted. Disagreements were resolved via an independent third investigator.

### 2.2 Inclusion and exclusion criteria

As the between-study heterogeneity is a known problem in the meta-analysis of prognostic marker studies, we used strict inclusion and exclusion criteria to limit the heterogeneity across studies. Our inclusion criteria were as follows: (1) the histological type of the tumors was confirmed as UTUC; (2) oncological outcomes of different tumor sizes were reported; and (3) prognostic value (hazard ratios [HRs] and 95% confidence intervals [95% CIs]) for tumor size were reported. Studies were excluded if those met one of the following criteria: (1) articles not published in English; (2) nonoriginal articles, such as review articles, commentaries, meeting abstracts, letters to the editor, or case reports; (3) laboratory studies, such as studies on cancer cell lines or animal models; and (4) studies that did not provide information on survival or could not offer sufficient data to acquire HRs and 95% CIs. When there was an overlap of patient cohorts between studies, the most recent or complete article was included in the analysis to avoid duplication of the same datasets.

### 2.3 Data extraction

Two investigators independently extracted the following information from the included articles: the first author’s name, publication year, recruitment country, period of patient recruitment, number of patients, study design, age, gender, tumor size, treatment methods, adjuvant therapy, follow-up duration, and oncological outcome. Subsequently, the HRs and 95% CIs of tumor size associated with each of the outcomes were retrieved. All discrepancies regarding data extraction were resolved by an independent third investigator.

### 2.4 Quality assessment of studies

The quality of the included studies was assessed using the Newcastle-Ottawa Scale (NOS) [13], which was recommended by the Cochrane Collaboration. The NOS assessed the quality of studies using a star system based on the following three domains: selection of the study groups (1–4 points), comparability of cohorts (1–2 points), and assessment of exposure and outcome (1–3 points), with total scores ranging from 0 (lowest) to 9 (highest). Studies with scores ≥8 were considered to have high quality, those with scores of 6–7 were considered to have intermediate quality, and those with scores <6 were considered to have low quality.

### 2.5 Statistical analyses

The endpoints of the present meta-analysis were OS, CSS, RFS, and IVR in UTUC patients. We extracted and combined HRs with the corresponding 95% CIs from every eligible study to analyze the prognostic value of tumor size. Heterogeneity between the studies was evaluated by Cochran’s Q test and I^2^ statistic. The random-effect model was applied to calculate the pooled HRs and 95% CIs if there was significant heterogeneity among the enrolled studies (I^2^ > 50% or *p* < 0.10). Alternatively, we used the fixed-effect model to perform cumulative analyses when no significant heterogeneity was found (I^2^ < 50% or *p* > 0.10). In addition, subgroup analyses, stratified by different study features, were conducted to evaluate the potential factors contributing to heterogeneity. Sensitivity analyses were performed by excluding studies with an NOS score of <7 or with unadjusted key confounding factors to assess the stability of the core results. The presence of publication bias was evaluated using the funnel plots. Statistical analyses were performed using Review Manager (Revman) 5.4 (The Nordic Cochrane Center, The Cochrane Collaboration, Copenhagen; 2014). All *p* values were two sided, and *p* < 0.05 was considered statistically significant.

## 3 Evidence synthesis

### 3.1 Search results

**Fig. 1:**
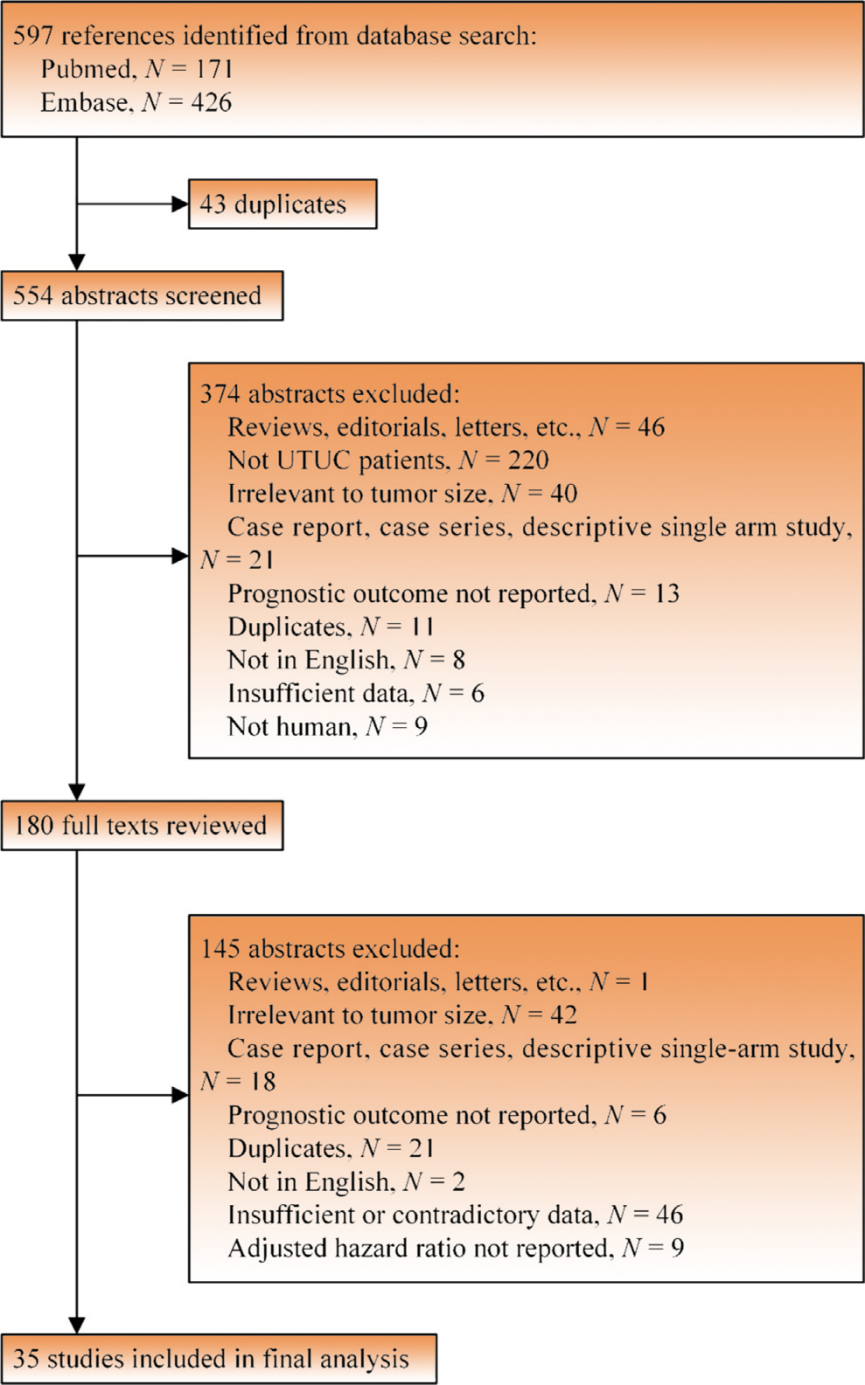
A flowchart of the study selection process. UTUC = upper tract urothelial carcinoma.

Following an initial electronic search, we identified 597 potentially eligible articles (171 in PubMed and 426 in Embase); finally, 35 articles published from 2006 to 2021, which met all the inclusion and exclusion criteria, were enrolled in this meta-analysis. Figure 1 presents a detailed flowchart of our selection process.

### 3.2 Study characteristics

The main characteristics of the 35 eligible studies are summarized in Table 1, Table 2. Briefly, a total of 32 292 participants were included in our meta-analysis, with a recruitment period from 2006 to 2021. The numbers of male and female participants were 18 927 and 13 365, respectively. All included studies had a retrospective study design. Among the 35 studies, 22 were conducted in Asia, seven in Europe, five in America, and one internationally. All patients in these studies had pathologically confirmed UTUC with different tumor sizes. The median age ranged from 65 to 75 yr, and the median follow-up periods ranged from 22 to 137 mo. The NOS scores ranged from 6 to 9, indicating moderate to high quality of the included studies.

**Table 1:** Characteristics of 35 studies included in the meta-analysis

Study	Year	Region	Recruitment	Study type	No. of patients	Oncological outcome	NOS
Cho et al. [26]	2006	Korea	1995–2004	RC	71	IVR	8
Wu et al. [10]	2007	Taiwan, China	1988–2003	RC	72	DFS, RFS	8
Simone et al. [16]	2009	Italy	1990–2006	RC	162	DFS, MFS	9
Pieras et al. [9]	2010	Spain	1990–2006	RC	79	IVR	9
Grasso et al. [27]	2012	USA	1996–2011	RC	160	OS, CSS, MFS	7
Holmäng and Johansson [28]	2014	Sweden	1971–1998	RC	614	IVR	9
Espiritu et al. [15]	2014	USA	1998–2012	RC	120	RFS	9
Shibing et al. [8]	2016	Mainland, China	2002–2012	RC	795	OS, CSS, RFS	9
Cui et al. [29]	2017	Mainland, China	2006–2013	RC	169	OS, CSS	9
Lee et al. [30]	2017	Korea	2000–2015	RC	760	OS, CSS, RFS, IVR	8
Yoo et al. [31]	2017	Korea	1999–2012	RC	418	OS, RFS	8
Toussi et al. [32]	2017	International	1995–2009	RC	372	CSS, RFS	8
Tseng et al. [33]	2017	Taiwan, China	2004–2015	RC	118	OS, CSS, RFS, MFS	8
Cho et al. [34]	2017	Korea	2004–2015	RC	1049	OS, CSS, RFS	8
Emamekhoo et al. [35]	2018	USA	1995–2014	RC	286	OS, PFS	9
Villa et al. [11]	2018	France	2003–2015	RC	92	PFS	9
Tan et al. [36]	2018	Mainland, China	2003–2015	RC	620	OS, CSS, RFS	8
Wang et al. [37]	2019	Mainland, China	2011–2017	RC	439	OS, IVR	9
Dong et al. [38]	2019	Mainland, China	2004–2014	RC	2731	OS, CSS	6
Li et al. [39]	2019	Mainland, China	1999–2015	RC	885	OS, CSS, PFS	6
Kang et al. [40]	2019	Korea	1994–2015	RC	338	CSS	6
Zhang et al. [41]	2020	Mainland, China	2007–2017	RC	568	IVR	8
Yang et al. [42]	2020	Mainland, China	2004–2015	RC	1768	OS, CSS	9
Nazzani et al. [43]	2020	Canada	2004–2014	RC	4266	CSM, OCM	9
Chen et al. [44]	2020	Mainland, China	2008–2018	RC	232	OS, CSS, RFS	8
Li et al. [45]	2020	Taiwan, China	2012–2016	RC	217	OS, RFS, IVR	9
Cheng et al. [46]	2021	Mainland, China	2006–2017	RC	398	CSS, RFS, DFS	9
Piraino et al. [47]	2020	USA	2004–2015	RC	8979	OS	8
Shvero et al. [48]	2021	Israel	2014–2019	RC	59	RFS, PFS	9
Hu and You [49]	2022	Mainland, China	2010–2015	RC	1979	OS, CSS	9
Sanguedolce et al. [50]	2021	Spain	2015–2019	RC	47	OS, BR, RFS, PFS	6
Li et al. [51]	2021	Mainland, China	1975–2016	RC	2576	CSD	9
Zhao et al. [52]	2021	Mainland, China	2008–2019	RC	316	OS, CSS	9
Chen et al. [53]	2022	Mainland, China	2010–2017	RC	195	OS, IRFS, CUTR	8
Milojevic et al. [54]	2021	Serbia	2000–2018	RC	342	CSS, RFS	7

BR = bladder recurrence; CSD = cancer-specific death; CSM = cancer-specific mortality; CSS = cancer-specific survival; CUTR = contralateral upper tract recurrence; DFS = disease-free survival; IRFS = IVR-free survival; IVR = intravesical recurrence; MFS = metastasis-free survival; NOS = Newcastle-Ottawa Scale; OCM = other-cause mortality; OS = overall survival; PFS = progression-free survival; RC = retrospective cohort; RFS = recurrence-free survival.

**Table 2:** Characteristics of patients in the included 35 studies

Study	Age (yr)	Gender (M/F)	Tumor size criteria (cm)	Measurement of tumor size	Treatment	Adjuvant therapy (%)	Follow-up (mo)
Cho et al. [26]	Mean: 65	48/23	3	NA	RNU	NA	Mean (range): 16.5 (3–28)
Wu et al. [10]	Mean ± SD: 66.7 ± 1.3	36/36	4	NA	RNU	12.5% (chemotherapy), 25% (radiotherapy)	Median (range): 26.5 (3–92)
Simone et al. [16]	NA	103/59	1, 3	Pathological specimens	RNU	NA	Median (range): 66 (58.4–196)
Pieras et al. [9]	Mean (range): 67 (65–69)	62/17	4	Pathological specimens	RNU	NA	Median (range): 71 (59–84)
Grasso et al. [27]	Median (range): 73 (45–93)	96/64	3	CT, MRI, ureteroscopic imaging	48.7% RNU, 1.3% SR, 50% URS	NA	Mean (range): 38.2 (1–185.3)
Holmäng and Johansson [28]	Median (range): 69 (25–92)	362/252	Continuous	Pathological specimens	54.4% RNU	NA	Median: 137
Espiritu et al. [15]	Median (range): 71 (64–78)	78/42	3	Pathological specimens	RNU	22.5% (chemotherapy)	Mean (range): 26.9 (8.5–36.8)
Shibing et al. [8]	Mean: 67	462/333	3	Pathological specimens	RNU	25.4% (chemotherapy)	Median (range): 32 (1–132)
Cui et al. [29]	Median (range): 66 (36–87)	107/62	4	Pathological specimens	87% RNU, 13% SR	91.7% (chemo/radiotherapy)	Mean ± SD: 53.7 ± 31.3
Lee et al. [30]	Mean: 65.5	561/199	Continuous	Pathological specimens	RNU	27.6% (chemotherapy)	Median (range): 45 (3–76)
Yoo et al. [31]	Mean ± SD: 63.8 ± 10.1	113/305	Continuous	Pathological specimens	RNU	NA	Mean: 69
Toussi et al. [32]	Median (range): 73.7 (65.4–79.5)	249/123	3	NA	RNU	NA	Median (range): 47 (16.4–101.4)
Tseng et al. [33]	Median (range): 70.5 (42–89)	47/71	Continuous	Pathological specimens	RNU	NA	Median (range): 26.9 (8.5–36.8)
Cho et al. [34]	Median (range): 68.5 (60.5–74.3)	759/290	Continuous	Pathological specimens	RNU	28.6% (chemotherapy)	Median (range): 40 (18.4–64.8)
Emamekhoo et al. [35]	Median: 72	190/96	5	NA	91% RNU, 8% SR, 1% URS	6% (chemotherapy)	Median (range): 39.5 (0.3–186)
Villa et al. [11]	Median (range): 71 (34–90)	62/30	1	Ureteroscopic imaging	URS	NA	Median (range): 52.4 (27.8–76.4)
Tan et al. [36]	Mean ± SD: 65.70 ± 11.35	355/265	3	Pathological specimens	RNU	41.1% (chemotherapy)	Median (range): 51 (1–168)
Wang et al. [37]	Mean: 66.7	236/203	3	Pathological specimens	RNU	NA	Mean (range): 62.5 (18–84)
Dong et al. [38]	Median (range): 72 (23±–96)	1557/1174	3, 5, 8	NA	RNU	12.6% (chemotherapy), 3.3% (radiotherapy)	Median: 31
Li et al. [39]	Median (range): 69 (61–75)	396/489	5	Radiological imaging	RNU	NA	Median (range): 61.0 (38–102)
Kang et al. [40]	Median (range): 65 (57–72)	245/93	Continuous	Pathological specimens	RNU	42.9% (chemotherapy)	Median (range): 31.5 (16.0–65.0)
Zhang et al. [41]	NA	294/274	2	CT	RNU	34.2% (chemotherapy)	NA
Yang et al. [42]	NA	996/772	1, 3	NA	NA	0%	NA
Nazzani et al. [43]	Median (range): 73 (64–80)	2501/1765	4	NA	RNU	NA	Median (range): 32 (14–63)
Chen et al. [44]	Median (range): 65 (58–73)	132/100	3	Ureteroscopic imaging, retrograde pyelography, CT, MRI	RNU	NA	Median (range): 39 (17–53)
Li et al. [45]	Median (range): 70 (34–90)	79/138	6.7	Pathological specimens	RNU	NA	Median (range): 42.0 (1.18–83.34)
Cheng et al. [46]	Median (range): 65.5 (20–92)	215/183	3	Pathological specimens	RNU	NA	Median (range): 55 (32–71)
Piraino et al. [47]	Mean ± SD: 72.4 ± 10.0	5510/3469	Continuous	NA	74.5% RNU, 25.5% SR	18.3% (chemotherapy)	NA
Shvero et al. [48]	Median (range): 70 (65–75)	41/18	1, 2, 3	CTU, MRU, retrograde pyelography, ureterorenoscopy	URS	NA	Median (range): 22 (11–41)
Hu and You [49]	Mean ± SD: 70.7 ± 11.2	949/1030	4.5, 6.7	NA	NA	NA	NA
Sanguedolce et al. [50]	Median (range): 75 (67–81)	35/12	Continuous	Ureteroscopic imaging	URS	NA	Median (range): 24 (17–44)
Li et al. [51]	Median: 71	1536/1040	2, 4	NA	NA	NA	NA
Zhao et al. [52]	Median (range): 69 (61–75)	205/111	Continuous	Pathological specimens	RNU	10.1% (chemotherapy)	Median (range): 43 (28–67)
Chen et al. [53]	Median (range): 68 (60–74)	120/75	3.1	CTU, MRI, ureteroscopic imaging	RNU	NA	Median: 46
Milojevic et al. [54]	Mean ± SD: 66.6 ± 8.9	190/152	3	Pathological specimens	RNU	23.4% (chemotherapy)	Median (range): 32.5 (6–154)

CT = computed tomography; CTU = computerized tomographic urography; F = female; M = male; MRI = magnetic resonance imaging; MRU = magnetic resonance urography; NA = not available; RNU = radical nephroureterectomy; SD = standard deviation; SR = segmental resection of ureter; URS = ureteroscopy.

### 3.3 Quality assessment of studies

All studies we analyzed scored from 6 to 9 using a nine-point scoring system. The result showed that most studies had good performance in sample selection and outcome, but failed in comparability. Assessment of study-specific quality scores from the NOS system is summarized in Supplementary Table 1.

### 3.4 Meta-analysis of survival outcomes

#### 3.4.1 Association of tumor size with OS

**Fig. 2:**
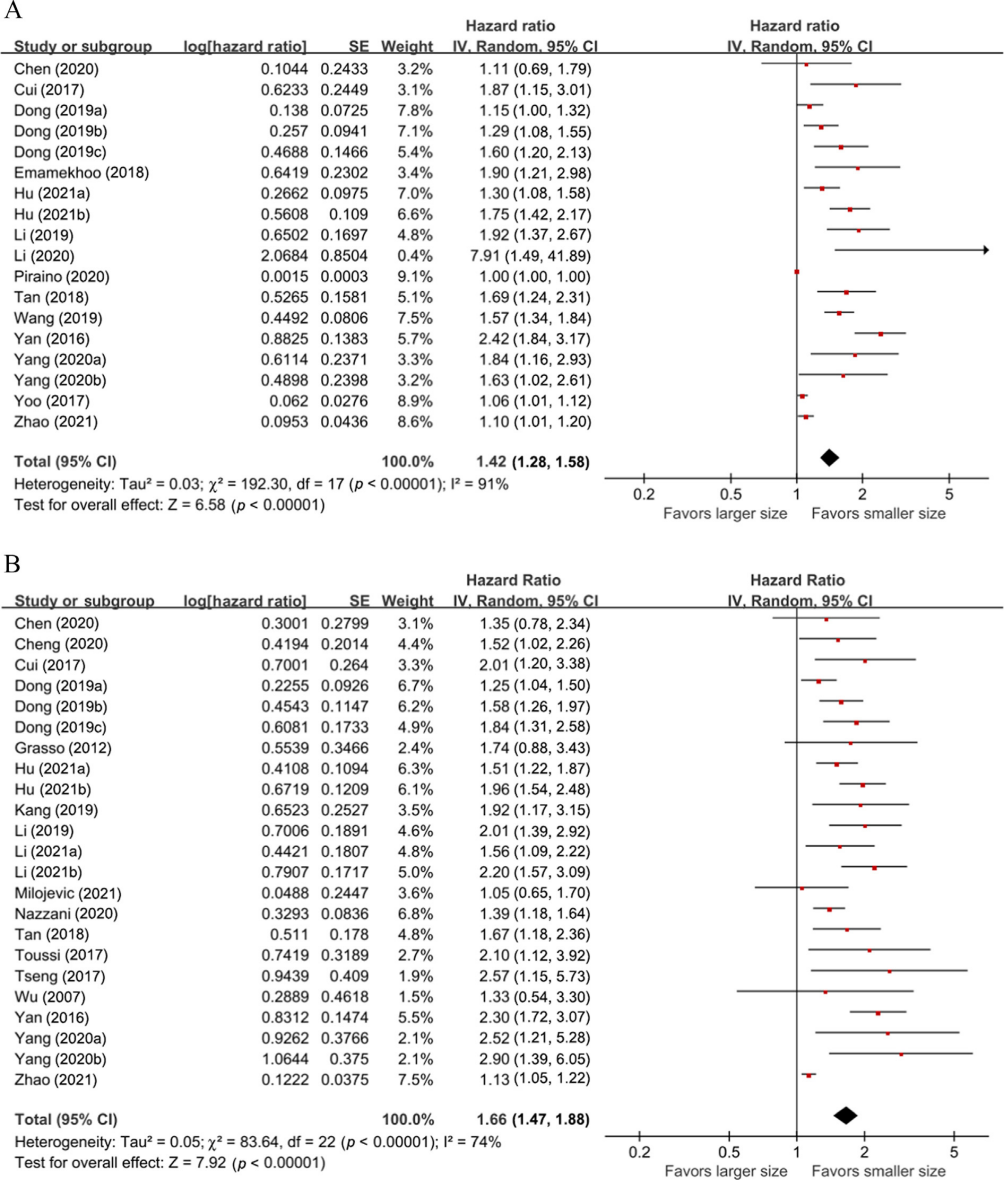

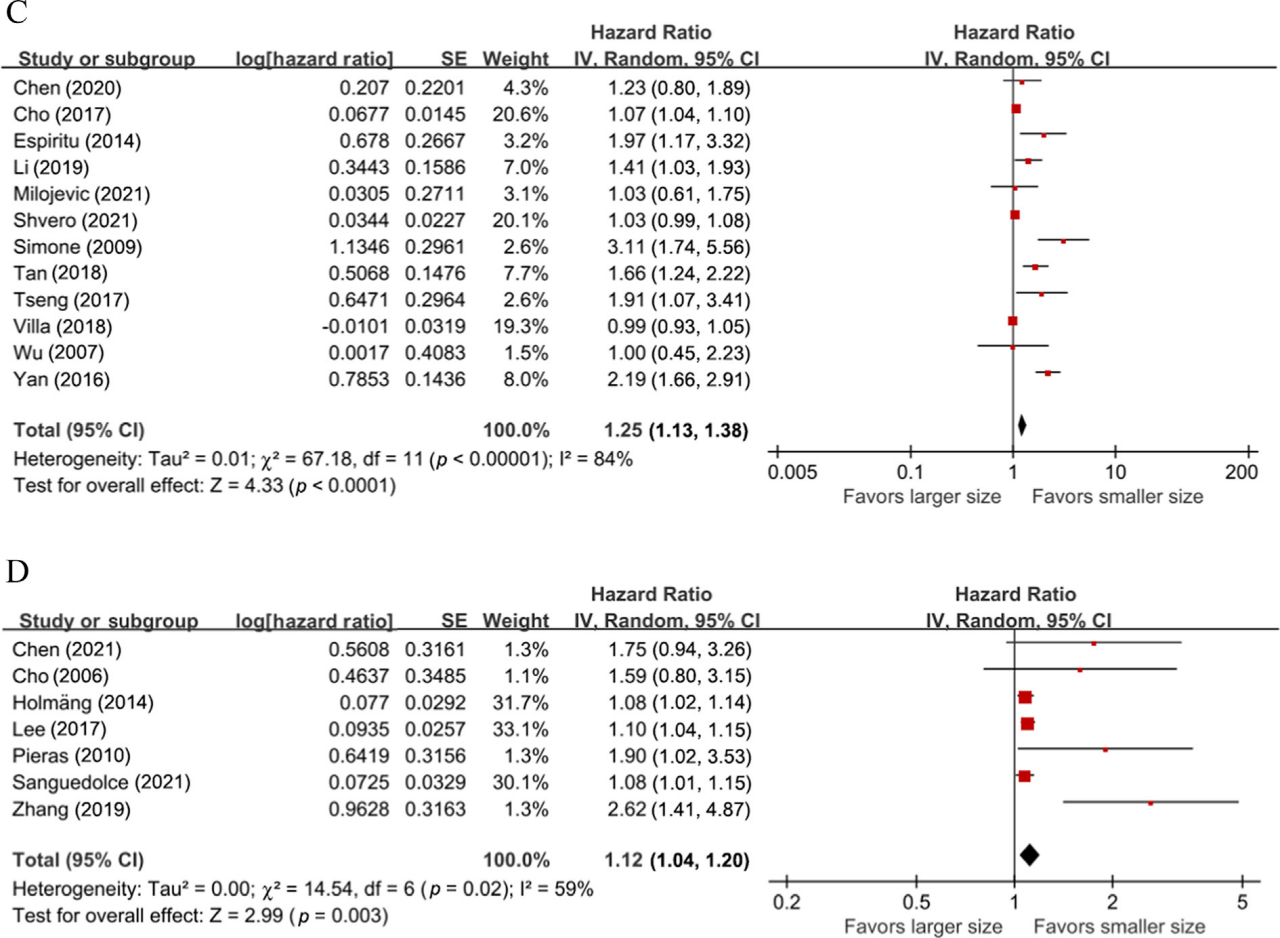
Forest plots showing the association between tumor size and survival outcomes in UTUC patients: (A) overall survival, (B) cancer-specific survival, (C) recurrence-free survival, and (D) intravesical recurrence. CI = confidence interval; df = degree of freedom; IV = inverse variance; SE = standard error; UTUC = upper tract urothelial carcinoma.

A total of 14 studies with 19 834 patients provided data on the association between tumor size and OS in patients with UTUC. The forest plot (Fig. 2A) showed that larger tumor size was significantly associated with shorter OS in UTUC patients (HR = 1.42, 95% CI = 1.28–1.58, z score = 6.58). The Cochrane Q test (chi-square = 192.30, *p* < 0.00001) and I^2^ test (91%) revealed significant heterogeneity.

#### 3.4.2 Association of tumor size with CSS

A total of 18 studies with 18 137 patients provided data on the association between tumor size and CSS in patients with UTUC. The forest plot (Fig. 2B) showed that larger tumor size was significantly associated with shorter CSS in UTUC patients (HR = 1.66, 95% CI = 1.47–1.88, z score = 7.92). The Cochrane Q test (chi-square = 83.64, *p* < 0.00001) and I^2^ test (74%) revealed significant heterogeneity.

#### 3.4.3 Association of tumor size with RFS

A total of 12 studies including 4546 patients provided data on the association between tumor size and RFS in patients with UTUC. The forest plot (Fig. 2C) showed that larger tumor size was significantly associated with shorter RFS in UTUC patients (HR = 1.25, 95% CI = 1.13–1.38, z score = 4.33). The Cochrane Q test (chi-square = 67.18, *p* < 0.00001) and I^2^ test (84%) revealed significant heterogeneity.

#### 3.4.4 Association of tumor size with IVR

A total of seven studies with 2334 patients provided data on the association between tumor size and IVR in patients with UTUC. The forest plot (Fig. 2D) showed that larger tumor size was significantly associated with higher IVR in UTUC patients (HR = 1.12, 95% CI = 1.04–1.20, z score = 2.99). The Cochrane Q test (chi-square = 14.54, *p* = 0.02) and I^2^ test (54%) revealed significant heterogeneity.

### 3.5 Subgroup analysis and sensitivity analysis

As statistically significant heterogeneity was detected among studies, further subgroup analyses were performed in the following cohorts: (1) cohorts receiving RNU, and not nephron-sparing surgery; (2) those receiving segmental resection of ureter; (3) those receiving adjuvant therapy including adjuvant chemotherapy and/or adjuvant radiotherapy; (4) those with negative lymph node; (5) those receiving no lymph node dissections; (6) those comparing tumor size ≥3 versus <3 cm, not treating tumor size as continuous variables; and (7) those in the regions of Asia, and Europe and America. The results suggested that segmental resection of ureter, whether receiving lymph node dissection, cutoff of tumor size, and region of cohorts might be the origin of potential heterogeneity as there was an obvious decrease of heterogeneity in these subgroups compared with the whole cohort (Table 3). In addition, tumor size was significantly associated with adverse prognosis in patients with UTUC in most subgroup analyses, except in the subgroup of Europe and America (OS and RFS), and the negative correlation is not affected by treatment modalities (whether patients receiving RNU or segmental resection of ureter, and whether receiving adjuvant therapy; Table 3). We also performed sensitivity analysis by excluding the studies with an NOS score of <7 or with unadjusted key confounding factors. The results indicated that the significant impact of tumor size on prognosis did not change when these studies were omitted (Supplementary Fig. 1), which confirmed the stability and reliability of our results.

**Table 3:** Subgroup analyses

Analysis specification	No. of studies	HR (95% CI)	Study heterogeneity		Effect model	*p* value
			I^2^ (%)	P_heterogeneity_		
Radical nephroureterectomy						
OS	9	1.42 (1.23–1.65)	87	<0.00001	Random	<0.00001
CSS	13	1.56 (1.35–1.80)	73	<0.00001	Random	<0.00001
RFS	10	1.55 (1.20–1.99)	84	<0.00001	Random	0.0008
IVR	5	1.62 (1.10–2.40)	71	0.009	Random	0.02
Segmental resection of ureter						
OS	2	1.88 (1.36–2.62)	0	0.96	Fixed	0.0002
CSS	2	1.91 (1.26–2.88)	0	0.74	Fixed	0.002
RFS	NA	NA	NA	NA	NA	NA
IVR	NA	NA	NA	NA	NA	NA
Adjuvant therapy						
OS	8	1.43 (1.23–1.66)	92	<0.00001	Random	<0.00001
CSS	9	1.60 (1.36–1.88)	81	<0.00001	Random	<0.00001
RFS	6	1.45 (1.03–2.03)	87	<0.00001	Random	0.03
IVR	1	1.59 (0.80–3.15)	NA	NA	Fixed	0.18
Negative lymph node						
OS	1	1.74 (1.25–2.42)	0	0.72	Fixed	0.001
CSS	4	1.77 (1.38–2.27)	52	0.06	Random	<0.00001
RFS	1	1.00 (0.45–2.23)	NA	NA	Random	1.00
IVR	1	1.90 (1.02–3.53)	NA	NA	Fixed	0.04
Without lymph node dissection						
OS	3	1.66 (0.93–2.94)	66	0.05	Random	0.08
CSS	2	1.57 (1.17–2.11)	0	0.52	Fixed	0.003
RFS	2	1.51 (1.19–1.92)	22	0.26	Fixed	0.0007
IVR	NA	NA	NA	NA	NA	NA
Tumor size (≥3 vs <3 cm)						
OS	4	1.69 (1.30–2.20)	72	0.01	Random	<0.0001
CSS	7	1.72 (1.47–2.02)	37	0.15	Fixed	<0.00001
RFS	6	1.74 (1.33–2.28)	61	0.02	Random	<0.0001
IVR	3	1.10 (1.05–1.16)	39	0.19	Fixed	0.0001
Region (Asia)						
OS	12	1.48 (1.30–1.68)	85	<0.00001	Random	<0.00001
CSS	14	1.71 (1.48–1.99)	78	<0.00001	Random	<0.00001
RFS	7	1.46 (1.10–1.92)	85	<0.00001	Random	0.008
IVR	4	1.58 (1.01–2.46)	72	0.01	Random	0.04
Region (Europe and America)						
OS	2	1.32 (0.71–2.47)	87	0.005	Random	0.38
CSS	3	1.37 (1.18–1.59)	0	0.43	Fixed	<0.0001
RFS	4	1.52 (0.90–2.58)	86	0.0001	Random	0.12
IVR	3	1.08 (1.04–1.13)	38	0.20	Fixed	0.0004

CI = confidence interval; CSS = cancer-specific survival; HR = hazard ratio; IVR = intravesical recurrence; NA = not available; OS = overall survival; RFS = recurrence-free survival.

### 3.6 Publication bias

Publication bias was evaluated using the funnel plots in our study. As presented in Supplementary Fig. 3, the shapes of the funnel plots indicated that there was no evident asymmetry, and thus, no evidence of publication bias was found in all indicators.

### 3.7 Discussion

Compared with bladder cancer, UTUC is usually in a more advanced stage at diagnosis and is associated with higher recurrence and progression rates [14]. It is necessary to identify high-risk patients for individualized treatment. Tumor size is the most common characteristic that can be acquired preoperatively and is an adverse predictor of oncological outcomes in most cancers; however, its prognostic value in UTUC is controversial.

The results of our study indicated that larger tumor size was significantly associated with poorer OS (HR = 1.42, 95% CI = 1.28–1.58, *p* < 0.00001), CSS (HR = 1.66, 95% CI = 1.47–1.88, *p* < 0.00001), RFS (HR = 1.25, 95% CI = 1.13–1.38, *p* < 0.0001), and IVR (HR = 1.12, 95% CI = 1.04–1.20, *p* = 0.003). To the best of our knowledge, the present study is the first meta-analysis to systematically evaluate these associations. The sample size of the current investigation is the largest to date in studies focusing on survival outcomes of UTUC, given the rarity of the disease. Our study is beneficial to clarify the controversial results about the prognostic value of tumor size in UTUC.

To address the heterogeneity of included studies, we performed several subgroup analyses. First, we analyzed the prognostic role tumor size in cohorts receiving only RNU and cohorts receiving only segmental resection of ureter. The rationale is that the 35 studies included in the primary analysis received a variety of local treatments such as RNU, segmental resection of ureter, or endourological resection. We assumed that the variety of treatments may be the source of heterogeneity. The subgroup analysis results confirmed that tumor size is a significantly poor survival predictor irrespective of whether patients received radical treatment or local resection. Different from the subgroup of receiving RNU, there is an obvious decrease of heterogeneity in the subgroup of receiving segmental resection of ureter compared with the whole cohort, which indicated that the treatment of segmental resection of ureter might be the potential source of heterogeneity. Considering that in addition to surgical treatment, adjuvant therapy may be another vital source of heterogeneity as well, we also performed the subgroup analysis in cohorts receiving adjuvant therapy, including adjuvant chemotherapy and/or adjuvant radiotherapy. The result is satisfactory, which is similar to primary analysis, although there was no decrease in heterogeneity. The above results suggest that the association of tumor size with adverse prognosis in patients with UTUC is not affected by treatment modalities. Subsequently, we excluded studies treating tumor size as a continuous variable, only focusing on studies comparing tumor size of ≥3 versus <3 cm, since 3 cm represented the median tumor diameter of the surgical specimens [15] and was the most common tumor size cutoff in relevant studies. Again, the results were consistent with our primary findings, and the heterogeneity decreased obviously in CSS and IVR, which suggested that the cutoff of tumor size may be the source of heterogeneity as well. In addition, we performed two subgroup analyses in cohorts with negative lymph nodes and without lymph node dissection. The heterogeneity of survival outcomes of patients with UTUC decreased significantly, suggesting that whether receiving lymph node dissection may be a potential source of heterogeneity, while the decrease of heterogeneity in the subgroup of negative lymph nodes may be due to a lack of literature. Finally, we also performed subgroup analyses based on the regional distribution, considering that most of the populations included in this meta-analysis were of Asian ethnicity. The subgroup of region (Europe and America) may be the other important source of heterogeneity with decreased heterogeneity in CSS and IVR. On the contrary, tumor size is not significant with worse OS and RFS in the subgroup of region (Europe and America), which indicated that the prognostic role of tumor size in UTUC could be affected by ethnicity. Future UTUC studies should encompass this feature as well as aforementioned potential source of heterogeneity into their reporting to guarantee the comparativeness between cohorts.

There are several theories to explain the biological mechanisms of the positive associations between tumor size and poor outcomes in UTUC. Tumor size has been identified to be associated with aggressive tumor biological behavior of UTUC in a number of studies, such as advanced tumor stage, lymph node metastasis, LVI, sessile tumor architecture, tumor necrosis, and tumor multifocality [8], [16], [17]. As advanced tumor stage has been deemed as one of the most important prognostic features in cancer, it is not difficult to understand why large tumor size heralds poor oncological outcomes in UTUC [18]. In addition, larger tumor size is more prone to LVI, which has been suggested to be a prerequisite for lymph node metastases [19]. It significantly increases the risk of disease recurrence, cancer-specific mortality, and overall mortality even after effective local treatment (RNU) [20]. Further, a larger tumor has a higher chance of extensive tumor necrosis. Simone et al. [16] reported that all the metastasis- and cancer-related deaths occurred in cases with extensive tumor necrosis (≥10% tumor area), which potentially explains the poor prognosis in large UTUC. Finally, Shibing et al. [8] found that patients with large tumor size were more likely to involve both the ureter and the renal pelvis, so they needed to receive open RNU instead of minimally invasive surgery, while open surgery was more likely to result in poorer surgical outcomes [21], [22].

Our findings have several clinical implications. First, our results provide strong evidence to support the role of tumor size in preoperative risk stratification. Though theoretically the depth of tumor invasion is a more important metric for risk stratification of urothelial carcinoma, it is difficult to determine the depth of tumor invasion in UTUC preoperatively. Ureteroscopic biopsy usually cannot get deep enough to the muscle layer, let alone whether ureteroscopic biopsy should be performed is still open to question. Preoperative radiographic examinations (ie, computed tomography and magnetic resonance imaging) are usually not indicative of tumor depth either. Thus, tumor size could serve as an ideal surrogate in preoperative risk stratification of UTUC, which is solidly supported by our results. More specifically, since tumor size impacts oncological outcomes of patients tremendously, it needs to be considered in the selection of treatment modalities. For example, although lymph node dissection is considered to be important in UTUC treatment, there has been no definite consensus regarding the clinical indications yet. Tumor size has the potential to be deemed as an objective variable for identifying lymph node dissection candidates in UTUC [8]. Similarly, an understanding of which patients are likely to have more aggressive disease based on tumor size may better guide the appropriate use of perioperative chemotherapy in UTUC. Given the common impairment of renal function after RNU [23], neoadjuvant chemotherapy (NAC) has been considered preferable to adjuvant chemotherapy and has become the gold standard [24]. However, the lack of enough pathological characteristics acquired preoperatively limits the appropriate selection of NAC candidates [25]. Therefore, identification of tumor size combined with other preoperative clinicopathological factors would be conducive to screening high-risk candidates suitable for administration of NAC.

The current study has several limitations that need to be acknowledged. First, all enrolled studies were retrospective in nature. The data extracted from those studies may lead to an inherent bias potentially. Second, most populations included in this meta-analysis were of Asian ethnicity, which might result in an ethnicity bias and limit the generalization of the results. Although we performed subgroup analyses based on the regional distribution of cohorts, the results showed inconsistency between the subgroup of Asia and the subgroup of Europe and America. Therefore, additional populations from other ethnicities are required to further validate the impact of tumor size on UTUC prognosis. Third, obvious heterogeneity among studies was observed, which limits the value of the results. Although the random-effect model takes into account the heterogeneity among studies, the conclusions reached in our meta-analysis should be interpreted with caution. Fourth, the vast majority of studies included only patients receiving RNU, which might lead to a selection bias as patients may be candidate to RNU for reasons of, namely, high-grade disease at biopsy and disease multifocality other than tumor size. Finally, in the case of UTUC, an increasing risk of poor prognosis with increasing tumor size is not necessarily controversial in any quality studies. It is more important to determine the cutoff size of high versus low risk, but our study could not answer the question. In addition, tumor size measurement is particularly inaccurate in UTUC, regardless of whether performed endoscopically or radiographically, and the cutoff for determining risk at 3 cm remains arbitrary. It is also unclear whether these sizes are of the index lesion or represent the cumulative size of all tumors in cases of multiple lesions. Therefore, further studies are supposed to be designed for determining the optimum cutoff of tumor size, which can then contribute to risk stratification guidelines.

In spite of these potential limitations, this meta-analysis has its own advantages. First, the sample size adopted was significantly larger than that in any individual study. The massive study population enhanced the statistical capabilities and ensured accurate risk estimations. In addition, all articles included in the final analysis were of high quality by NOS scores, which increase the reliability of the pooled results.

## 4 Conclusions

In summary, this meta-analysis revealed that tumor size is associated with an increased risk of overall and cancer-specific mortality and disease recurrence in UTUC. Integration of tumor size with other prognostic indicators may help in risk stratification and individualized treatment options. However, given the study limitations including heterogeneity and retrospective nature of the primary data, these results need to be confirmed further by adequately designed prospective studies with larger populations to provide a better conclusion.

  ***Author contributions:*** Jian Lu had full access to all the data in the study and takes responsibility for the integrity of the data and the accuracy of the data analysis.

*Study concept and design*: Lu, L. Ma, Huang.

*Acquisition of data*: R. Ma, Liu, Cheng, Zhou, Pan, Yang, Xia, Zhu, J. He, W. He.

*Analysis and interpretation of data*: Cheng, Tao.

*Drafting of the manuscript*: Liu.

*Critical revision of the manuscript for important intellectual content*: R. Ma, Lu.

*Statistical analysis*: Cheng, Tao.

*Obtaining funding*: None.

*Administrative, technical, or material support*: None.

*Supervision*: Bi, Yang, Xia, Zhu, J. He, W. He, Wang, Huang.

*Other*: None.

  ***Financial disclosures:*** Jian Lu certifies that all conflicts of interest, including specific financial interests and relationships and affiliations relevant to the subject matter or materials discussed in the manuscript (eg, employment/affiliation, grants or funding, consultancies, honoraria, stock ownership or options, expert testimony, royalties, or patents filed, received, or pending), are the following: None.

  ***Funding/Support and role of the sponsor:*** None.

## References

[1] Siegel R.L., Miller K.D., Jemal A. (2020). Cancer statistics, 2020. CA Cancer J Clin.

[2] Rouprêt M., Babjuk M., Burger M. (2021). European Association of Urology guidelines on upper urinary tract urothelial carcinoma: 2020 update. Eur Urol.

[3] Margulis V., Shariat S.F., Matin S.F. (2009). Outcomes of radical nephroureterectomy: a series from the Upper Tract Urothelial Carcinoma Collaboration. Cancer.

[4] Ploussard G., Xylinas E., Lotan Y. (2015). Conditional survival after radical nephroureterectomy for upper tract carcinoma. Eur Urol.

[5] Cha E.K., Shariat S.F., Kormaksson M. (2012). Predicting clinical outcomes after radical nephroureterectomy for upper tract urothelial carcinoma. Eur Urol.

[6] Seisen T., Granger B., Colin P. (2015). A systematic review and meta-analysis of clinicopathologic factors linked to intravesical recurrence after radical nephroureterectomy to treat upper tract urothelial carcinoma. Eur Urol.

[7] Cheng L., Montironi R., Davidson D.D., Lopez-Beltran A. (2009). Staging and reporting of urothelial carcinoma of the urinary bladder. Mod Pathol.

[8] Shibing Y., Liangren L., Qiang W. (2016). Impact of tumor size on prognosis of upper urinary tract urothelial carcinoma after radical nephroureterectomy: a multi-institutional analysis of 795 cases. BJU Int.

[9] Pieras E., Frontera G., Ruiz X., Vicens A., Ozonas M., Pizá P. (2010). Concomitant carcinoma in situ and tumor size are prognostic factors for bladder recurrence after nephroureterectomy for upper tract transitional cell carcinoma. BJU Int.

[10] Wu C.F., Pang S.T., Chen C.S., Chuang C.K., Chen Y., Lin P.Y. (2007). The impact factors on prognosis of patients with pT3 upper urinary tract transitional cell carcinoma. J Urol.

[11] Villa L., Haddad M., Capitanio U. (2018). Which patients with upper tract urothelial carcinoma can be safely treated with flexible ureteroscopy with holmium:YAG laser photoablation? Long-term results from a high-volume institution. J Urol.

[12] Liberati A., Altman D.G., Tetzlaff J. (2009). The PRISMA statement for reporting systematic reviews and meta-analyses of studies that evaluate health care interventions: explanation and elaboration. PLoS Med.

[13] Stang A. (2010). Critical evaluation of the Newcastle-Ottawa scale for the assessment of the quality of nonrandomized studies in meta-analyses. Eur J Epidemiol.

[14] Kim M., Jeong C.W., Kwak C., Kim H.H., Ku J.H. (2015). Are urothelial carcinomas of the upper urinary tract a distinct entity from urothelial carcinomas of the urinary bladder? Behavior of urothelial carcinoma after radical surgery with respect to anatomical location: a case control study. BMC Cancer.

[15] Espiritu P.N., Sverrisson E.F., Sexton W.J. (2014). Effect of tumor size on recurrence-free survival of upper tract urothelial carcinoma following surgical resection. Urol Oncol.

[16] Simone G., Papalia R., Loreto A., Leonardo C., Sentinelli S., Gallucci M. (2009). Independent prognostic value of tumour diameter and tumour necrosis in upper urinary tract urothelial carcinoma. BJU Int.

[17] Wu Y., Dong Q., Liu L., Han P., Wei Q. (2014). The impact of tumor location and multifocality on prognosis for patients with upper tract urothelial carcinoma: a meta-analysis. Sci Rep.

[18] Su X., Fang D., Li X. (2016). The influence of tumor size on oncologic outcomes for patients with upper tract urothelial carcinoma after radical nephroureterectomy. Biomed Res Int.

[19] Novara G., Matsumoto K., Kassouf W. (2010). Prognostic role of lymphovascular invasion in patients with urothelial carcinoma of the upper urinary tract: an international validation study. Eur Urol.

[20] Liu W., Sun L., Guan F., Wang F., Zhang G. (2019). Prognostic value of lymphovascular invasion in upper urinary tract urothelial carcinoma after radical nephroureterectomy: a systematic review and meta-analysis. Dis Markers.

[21] Hanna N., Sun M., Trinh Q.D. (2012). Propensity-score-matched comparison of perioperative outcomes between open and laparoscopic nephroureterectomy: a national series. Eur Urol.

[22] Simone G., Papalia R., Guaglianone S. (2009). Laparoscopic versus open nephroureterectomy: perioperative and oncologic outcomes from a randomized prospective study. Eur Urol.

[23] Yafi F.A., Tanguay S., Rendon R. (2014). Adjuvant chemotherapy for upper-tract urothelial carcinoma treated with nephroureterectomy: assessment of adequate renal function and influence on outcome. Urol Oncol.

[24] Leow J.J., Martin-Doyle W., Fay A.P., Choueiri T.K., Chang S.L., Bellmunt J. (2014). A systematic review and meta-analysis of adjuvant and neoadjuvant chemotherapy for upper tract urothelial carcinoma. Eur Urol.

[25] Porten S., Siefker-Radtke A.O., Xiao L. (2014). Neoadjuvant chemotherapy improves survival of patients with upper tract urothelial carcinoma. Cancer.

[26] Cho D.H., Kim J.S., Kim H.T., Yoo E.S., Kwon T.G., Kim B.W. (2006). Risk factors for subsequent bladder cancer recurrence following radical surgery for upper urinary tract urothelial cancer. Korean J Urol.

[27] Grasso M., Fishman A.I., Cohen J., Alexander B. (2012). Ureteroscopic and extirpative treatment of upper urinary tract urothelial carcinoma: a 15-year comprehensive review of 160 consecutive patients. BJU Int.

[28] Holmäng S., Johansson S.L. (2014). Long-term follow-up of patients with tumours of the renal pelvis and ureter: how often is a bladder tumour diagnosed after five tumour-free years?. Scand J Urol.

[29] Cui J., Yu M., Zhang N. (2017). Prognostic scores based on the preoperative plasma fibrinogen and serum albumin level as a prognostic factor in patients with upper urinary tract urothelial carcinoma. Oncotarget.

[30] Lee C.H., Ku J.Y., Jeong C.W. (2017). Predictors for intravesical recurrence following radical nephroureterectomy for upper tract urothelial carcinoma: a national multicenter analysis. Clin Genitourin Cancer.

[31] Yoo S., You D., Jeong I.G. (2017). Does lymph node dissection during nephroureterectomy affect oncological outcomes in upper tract urothelial carcinoma patients without suspicious lymph node metastasis on preoperative imaging studies?. World J Urol.

[32] Toussi A., Miest T., Boorjian S. (2017). Oncological outcomes comparing intravesical and extravesical bladder cuff excision following radical nephroureterectomy for upper tract urothelial carcinoma. J Urol.

[33] Tseng J.S., Chiu A.W., Chen M. (2017). Oncological outcomes of laparoscopic nephroureterectomy with pluck method for distal ureter resection. Urol Sci.

[34] Cho Y.H., Hwang J.E., Chung H.S. (2017). The De Ritis (aspartate transaminase/alanine transaminase) ratio as a predictor of oncological outcomes in patients after surgery for upper urinary tract urothelial carcinoma. Int Urol Nephrol.

[35] Emamekhoo H., Dhillon P., Gopalakrishnan D. (2018). Prognostic factors and risk stratification in invasive upper tract urothelial carcinoma. Clin Genitourin Cancer.

[36] Tan P., Xie N., Liao H. (2018). Prognostic impact of preoperative anemia on upper tract urothelial carcinoma. Medicine (Baltimore).

[37] Wang Q., Zhang T., Wu J. (2019). Prognosis and risk factors of patients with upper urinary tract urothelial carcinoma and postoperative recurrence of bladder cancer in central China. BMC Urol.

[38] Dong F., Xu T., Wang X. (2019). Lymph node dissection could bring survival benefits to patients diagnosed with clinically node-negative upper urinary tract urothelial cancer: a population-based, propensity score-matched study. Int J Clin Oncol.

[39] Li Y., Fang D., Bao Z. (2019). High aspartate transaminase/alanine transaminase ratio predicts poor prognosis in patients with localized upper tract urothelial cancer: a propensity score-matched study in a large Chinese center. Onco Targets Ther.

[40] Kang M., Yoo H., Kim K. (2019). Role of adjuvant chemotherapy in advanced stage upper urinary tract urothelial carcinoma after radical nephroureterectomy: competing risk analysis after propensity score-matching. J Cancer.

[41] Zhang X., Bu R., Liu Z., Wu B., Bai S. (2020). Development and validation of a model for predicting intravesical recurrence in organ-confined upper urinary tract urothelial carcinoma patients after radical nephroureterectomy: a retrospective study in one center with long-term follow-up. Pathol Oncol Res.

[42] Yang T., Zhang N., Yang B., He D., Fan J., Fan J. (2020). Preintervention risk stratification of renal pelvic cancer and ureteral cancer should differ. Investig Clin Urol.

[43] Nazzani S., Preisser F., Mazzone E. (2020). Nephroureterectomy with or without bladder cuff excision for localized urothelial carcinoma of the renal pelvis. Eur Urol Focus.

[44] Chen X., Ji H., Wang J. (2020). Prognostic value of the preoperative plasma D-dimer levels in patients with upper tract urothelial carcinoma in a retrospective cohort study. Onco Targets Ther.

[45] Li Y.R., Yu K.J., Chang Y.H. (2020). Predictors of intravesical recurrence after radical nephroureterectomy and prognosis in patients with upper tract urothelial carcinoma. Cancer Manag Res.

[46] Cheng S., Zhong W., Xia K. (2021). Prognostic role of stromal tumor-infiltrating lymphocytes in locally advanced upper tract urothelial carcinoma: A retrospective multicenter study (TSU-02 study). Oncoimmunology.

[47] Piraino J.A., Snow Z.A., Edwards D.C., Hager S., McGreen B.H., Diorio G.J. (2020). Nephroureterectomy vs. segmental ureterectomy of clinically localized, high–grade, urothelial carcinoma of the ureter: practice patterns and outcomes. Urol Oncol.

[48] Shvero A., Abu-Ghanem Y., Laufer M. (2021). Endoscopic treatment for large multifocal upper tract urothelial carcinoma. J Urol.

[49] Hu T., You S. (2022). Overall and cancer-specific survival in patients with renal pelvic transitional cell carcinoma: a population-based study. Front Med (Lausanne).

[50] Sanguedolce F., Fontana M., Turco M. (2021). Endoscopic management of upper urinary tract urothelial carcinoma: oncologic outcomes and prognostic factors in a contemporary cohort. J Endourol.

[51] Li C., Han D., Huang Q. (2021). Competing-risks nomogram for predicting cancer-specific death in upper tract urothelial carcinoma: a population-based analysis. BMJ Open.

[52] Zhao F., Qi N., Zhang C. (2021). Impact of surgical wait time on survival in patients with upper urinary tract urothelial carcinoma with hydronephrosis. Front Oncol.

[53] Chen H., Wang M., Weng T. (2022). Prognostic analysis of diagnostic ureteroscopic biopsy for intravesical recurrence of upper urinary tract urothelial carcinoma. Urol Int.

[54] Milojevic B., Bumbasirevic U., Santric V. (2021). Prognostic significance of tumor multifocality on outcomes in patients with upper tract urothelial carcinoma after radical nephroureterectomy: a cohort study. Curr Probl Cancer.

