# Supplementary material for: Prognostic Value of Tumor Size in Patients with Upper Tract Urothelial Carcinoma: A Systematic Review and Meta-analysis
Source: Eur Urol Open Sci. 2022 Jun 28;42:19–29. doi: 10.1016/j.euros.2022.06.001 (PMC9244730; doi:10.1016/j.euros.2022.06.001)
Supplement: Supplementary data 1 [file mmc1.docx]

**Supplementary material**

A


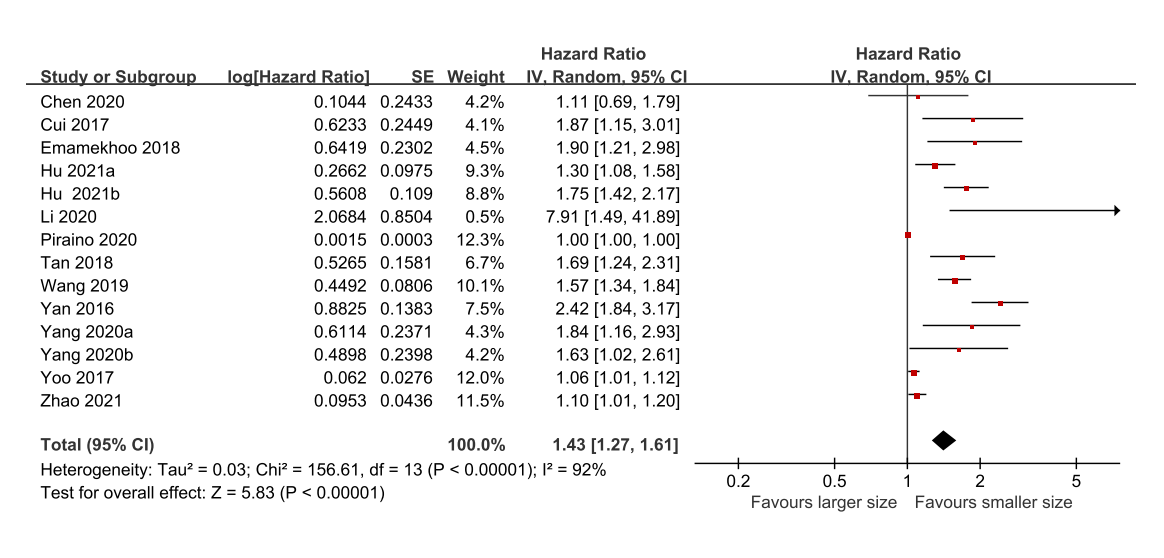


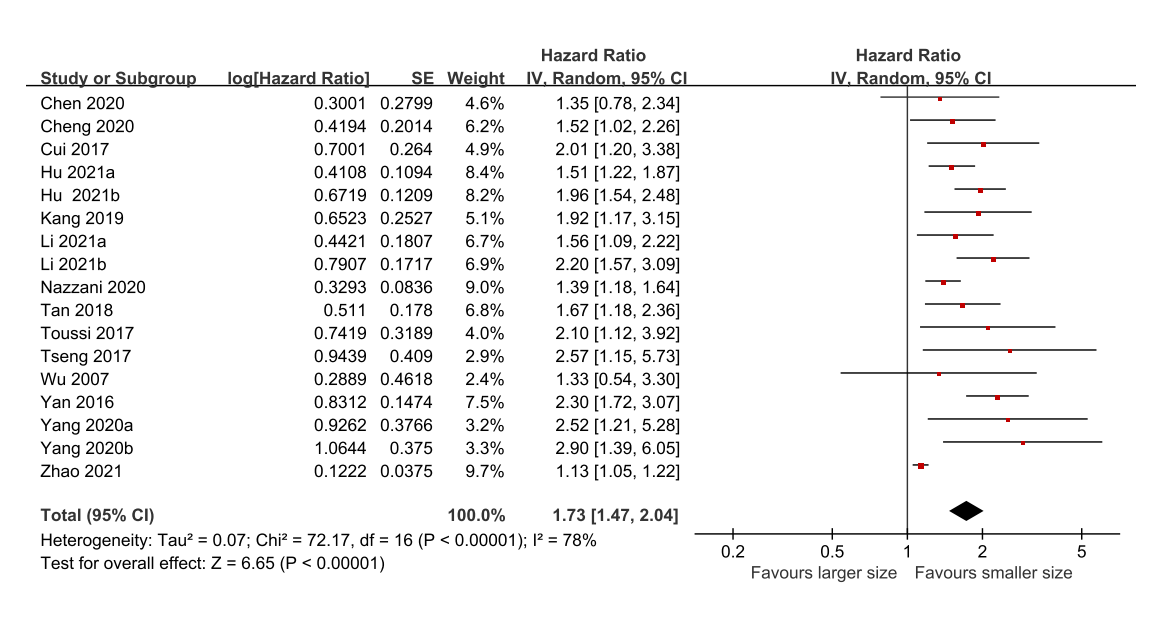


B


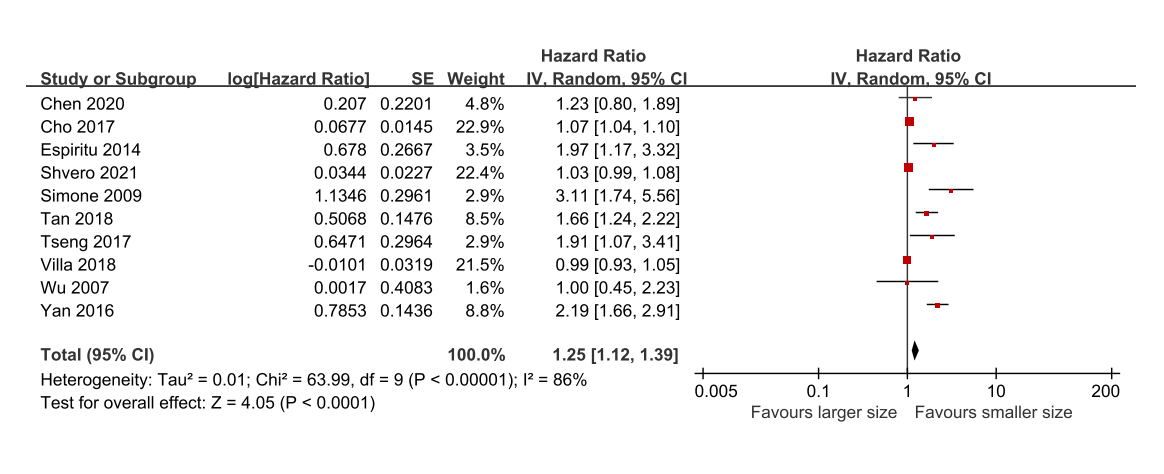


C


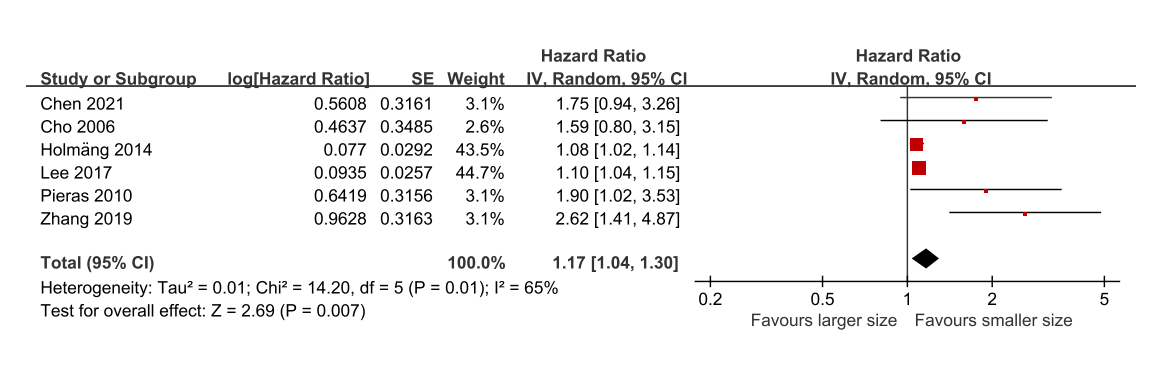


D

Supplementary Fig. 1 - Forest plots showing the association between tumor size and survival outcomes after sensitivity analysis. (A) overall survival; (B) cancer-specific survival; (C) recurrence-free survival; (D) intravesical recurrence. CI=confidence interval; SE=standard error.

A


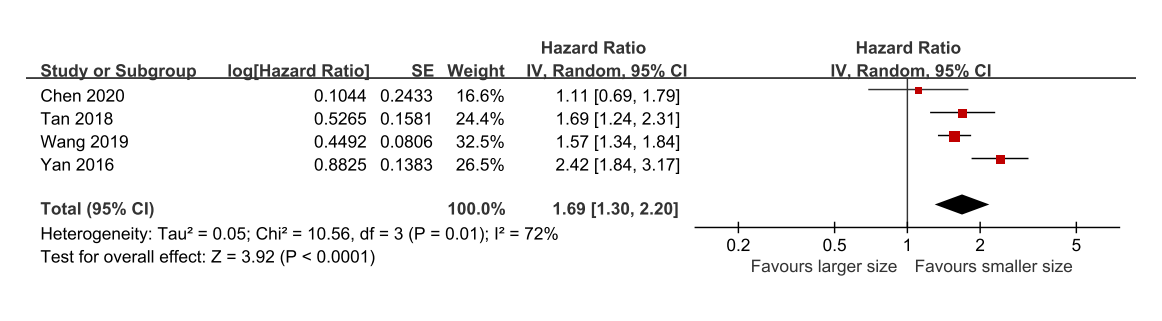


B


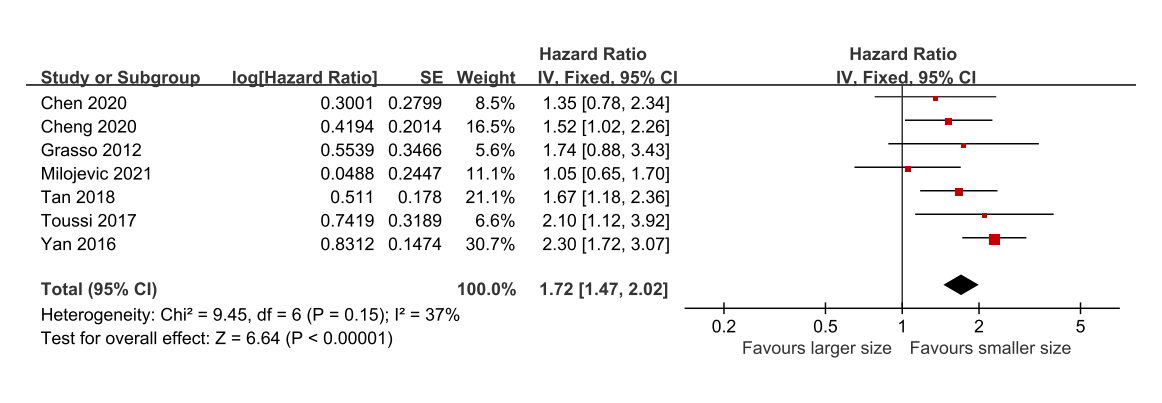


C


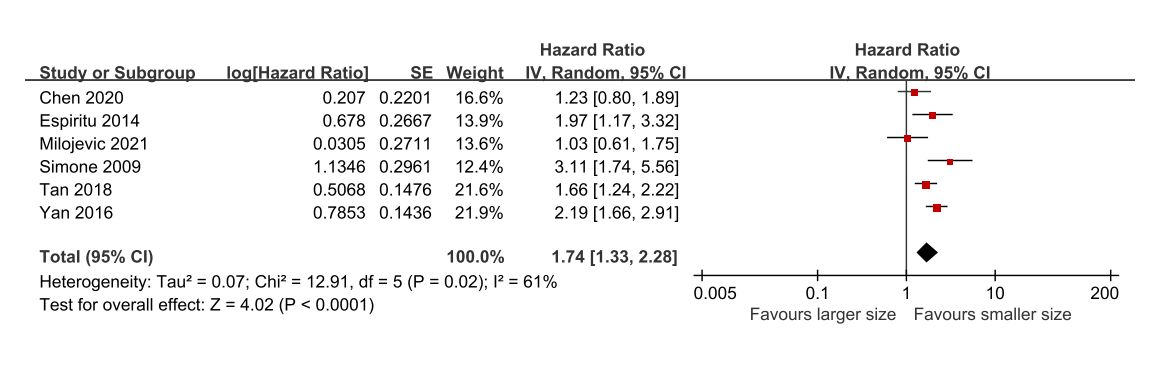


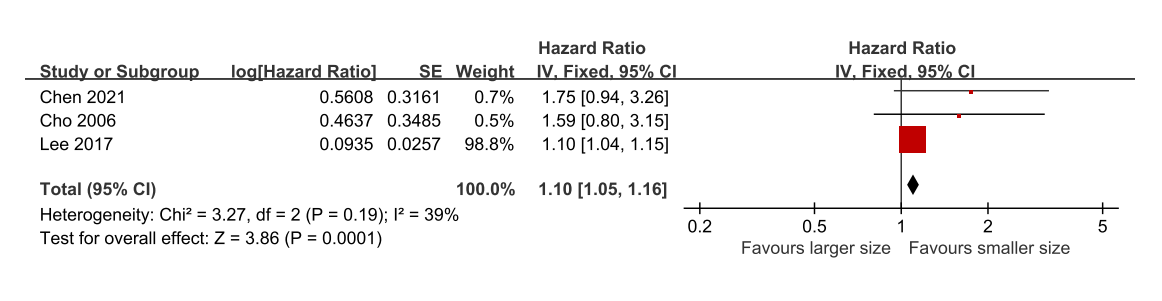


D

Supplementary Fig. 2 - Forest plots of the studies using 3cm as cut-of value showing the association between tumor size and survival outcomes. (A) overall survival; (B) cancer-specific survival; (C) recurrence-free survival; (D) intravesical recurrence. CI=confidence interval; SE=standard error.

A

| 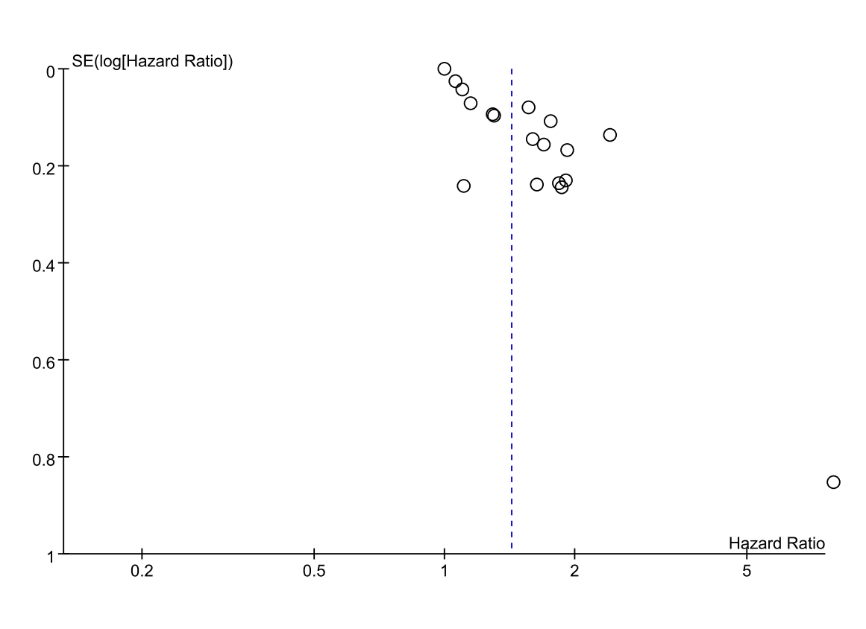  C | 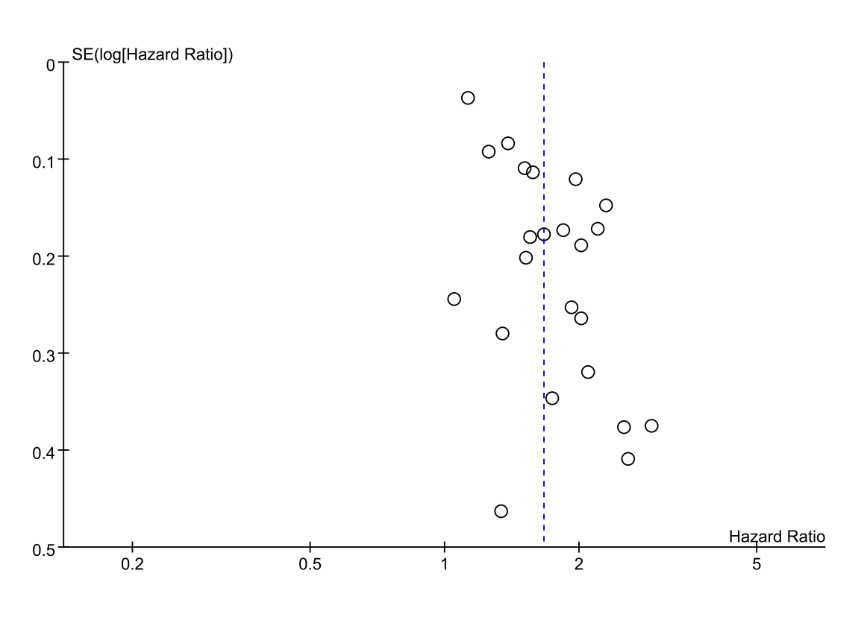  D  B |
| --- | --- |
| 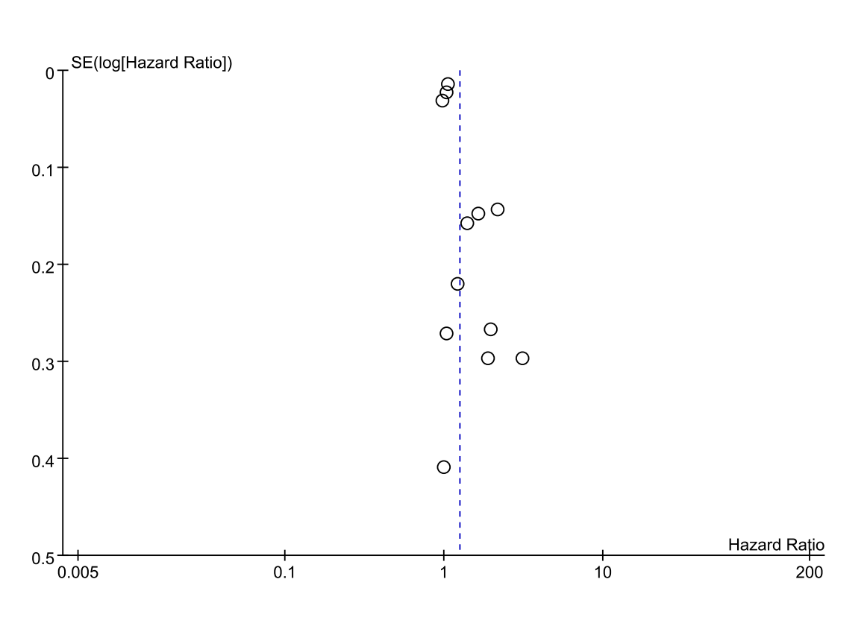 | 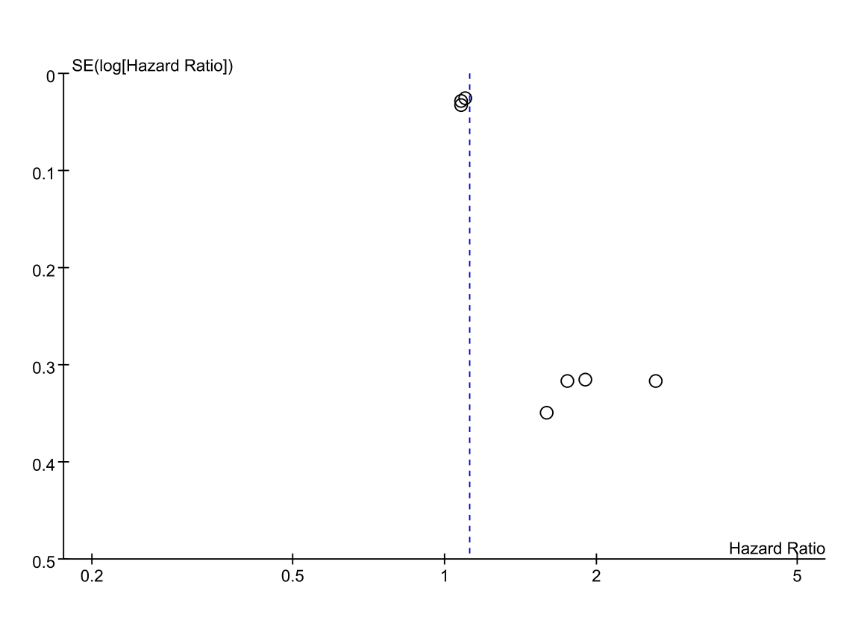 |

A

Supplementary Fig. 3 - Funnel plots of the assessment of potential publication bias in studies: (A) overall survival; (B) cancer-specific survival; (C) recurrence-free survival; (D) intravesical recurrence.

**Supplementary Table 1 - Quality of studies included in the meta-analysis**

| **Study** | **Newcastle–Ottawa Quality Assessment Scale** | | | | | | | | **Score** |
| --- | --- | --- | --- | --- | --- | --- | --- | --- | --- |
|  | **Selection** | | | | **Comparability** | **Outcome** | | |  |
|  | Q1 | Q2 | Q3 | Q4 | Q5 | Q6 | Q7 | Q8 |  |
| Cho DH et al. [14] | 1 | 1 | 1 | 1 | 2 | 1 | 1 | 0 | 8 |
| Wu CF et al. [10] | 1 | 1 | 1 | 1 | 2 | 1 | 1 | 0 | 8 |
| Simone G et al. [15] | 1 | 1 | 1 | 1 | 2 | 1 | 1 | 1 | 9 |
| Pieras E et al. [9] | 1 | 1 | 1 | 1 | 2 | 1 | 1 | 1 | 9 |
| Grasso M et al. [16] | 1 | 1 | 1 | 1 | 0 | 1 | 1 | 1 | 7 |
| Holmang S et al. [17] | 1 | 1 | 1 | 1 | 2 | 1 | 1 | 1 | 9 |
| Espiritu PN et al. [18] | 1 | 1 | 1 | 1 | 2 | 1 | 1 | 1 | 9 |
| Shibing Y et al. [8] | 1 | 1 | 1 | 1 | 2 | 1 | 1 | 1 | 9 |
| Cui J et al. [19] | 1 | 1 | 1 | 1 | 2 | 1 | 1 | 1 | 9 |
| Lee CH et al. [20] | 1 | 1 | 1 | 1 | 1 | 1 | 1 | 1 | 8 |
| Yoo S et al. [21] | 1 | 1 | 1 | 1 | 1 | 1 | 1 | 1 | 8 |
| Toussi A et al. [22] | 1 | 1 | 1 | 1 | 1 | 1 | 1 | 1 | 8 |
| Tseng JS et al. [23] | 1 | 1 | 1 | 1 | 1 | 1 | 1 | 1 | 8 |
| Cho YH et al. [24] | 1 | 1 | 1 | 1 | 1 | 1 | 1 | 1 | 8 |
| Emamekhoo H et al. [25] | 1 | 1 | 1 | 1 | 2 | 1 | 1 | 1 | 9 |
| Villa L et al. [11] | 1 | 1 | 1 | 1 | 2 | 1 | 1 | 1 | 9 |
| Tan P et al. [26] | 1 | 1 | 1 | 1 | 1 | 1 | 1 | 1 | 8 |
| Wang Q et al. [27] | 1 | 1 | 1 | 1 | 2 | 1 | 1 | 1 | 9 |
| Dong F et al. [28] | 1 | 1 | 1 | 1 | 2 | 0 | 0 | 0 | 6 |
| Li Y et al. [29] | 1 | 1 | 1 | 1 | 1 | 0 | 1 | 0 | 6 |
| Kang M et al. [30] | 1 | 1 | 1 | 1 | 1 | 0 | 1 | 0 | 6 |
| Zhang X et al. [31] | 1 | 1 | 1 | 1 | 1 | 1 | 1 | 1 | 8 |
| Yang T et al. [32] | 1 | 1 | 1 | 1 | 2 | 1 | 1 | 1 | 9 |
| Nazzani S et al. [33] | 1 | 1 | 1 | 1 | 2 | 1 | 1 | 1 | 9 |
| Chen X et al. [34] | 1 | 1 | 1 | 1 | 1 | 1 | 1 | 1 | 8 |
| Li YR et al. [35] | 1 | 1 | 1 | 1 | 2 | 1 | 1 | 1 | 9 |
| Cheng S et al. [36] | 1 | 1 | 1 | 1 | 2 | 1 | 1 | 1 | 9 |
| Piraino JA et al. [37] | 1 | 1 | 1 | 1 | 1 | 1 | 1 | 1 | 8 |
| Shvero A et al. [38] | 1 | 1 | 1 | 1 | 2 | 1 | 1 | 1 | 9 |
| Hu T et al. [39] | 1 | 1 | 1 | 1 | 2 | 1 | 1 | 1 | 9 |
| Sanguedolce F et al. [40] | 1 | 1 | 1 | 1 | 1 | 1 | 0 | 0 | 6 |
| Li C et al. [41] | 1 | 1 | 1 | 1 | 2 | 1 | 1 | 1 | 9 |
| Zhao F et al. [42] | 1 | 1 | 1 | 1 | 2 | 1 | 1 | 1 | 9 |
| Chen H et al. [43] | 1 | 1 | 1 | 1 | 1 | 1 | 1 | 1 | 8 |
| Milojevic B et al. [44] | 1 | 1 | 1 | 1 | 1 | 1 | 0 | 1 | 7 |

**Notes:** The data is presented using the Newcastle-Ottawa Quality Assessment Scale. Q1: the exposed cohort was truly or somewhat representative? Q2: the non-exposed cohort was drawn from the same community as the exposed cohort? Q3: exposure was ascertained by secure record or structured interview? Q4: outcome of interest was not present at start of study? Q5: on the basis of the design or analysis, cohorts had comparability? (controls for the most important factor? controls for any additional factor?) Q6: outcome was independent blind assessment or record linkage? Q7: follow-up was long enough for outcomes to occur? Q8: follow-up of cohorts was adequate? A study can be awarded a maximum of one point for each numbered item within the selection and outcome categories. A maximum of two points can be given for comparability.

Q=question.
